# Supplementary material for: Intraperitoneal microbial contamination drives post-surgical peritoneal adhesions by mesothelial EGFR-signaling
Source: Nat Commun. 2021 Dec 16;12:7316. doi: 10.1038/s41467-021-27612-x (PMC8677808; doi:10.1038/s41467-021-27612-x)
Supplement: Supplementary file 1 — Supplementary Information [file 41467_2021_27612_MOESM1_ESM.pdf]

Supplementary Information for "Intraperitoneal microbial contamination drives post-surgical peritoneal adhesions by mesothelial EGFR-signaling"

Joel Zindel[1,2*] Jonas Mittner[1], Julia Bayer[1], Simon L. April-Monn[3], Andreas Kohler[1], Ysbrand Nusse[2], Michel Dosch[1], Isabel Büchi[1], Daniel Sanchez-Taltavull[1], Heather Dawson[3], Mercedes Gomez de Agüero[1], Kinji Asahina[4,5], Paul Kubes[2], Andrew J. Macpherson[1], Deborah Stroka[1#] and Daniel Candinas[1#]

[1] Department of Visceral Surgery and Medicine, Inselspital, Bern University Hospital, University of Bern, Switzerland

[2] Department of Pharmacology and Physiology and Snyder Institute for Chronic Diseases and Department of Microbiology, Immunology & Infectious Diseases, Cumming School of Medicine, University of Calgary, Calgary, Alberta, Canada

[3] Clinical Pathology Division and Translational Research Unit, Institute of Pathology, University of Bern, Bern, Switzerland

[4] Southern California Research Center for Alcoholic Liver and Pancreatic Diseases and Cirrhosis and Department of Pathology, Keck School of Medicine of the University of Southern California, Los Angeles, CA, USA.

[5] Central Research Laboratory, Shiga University of Medical Science, Otsu, Shiga, Japan.

*To whom correspondence should be addressed to: joel.zindel@dbmr.unibe.ch

# These authors contributed equally

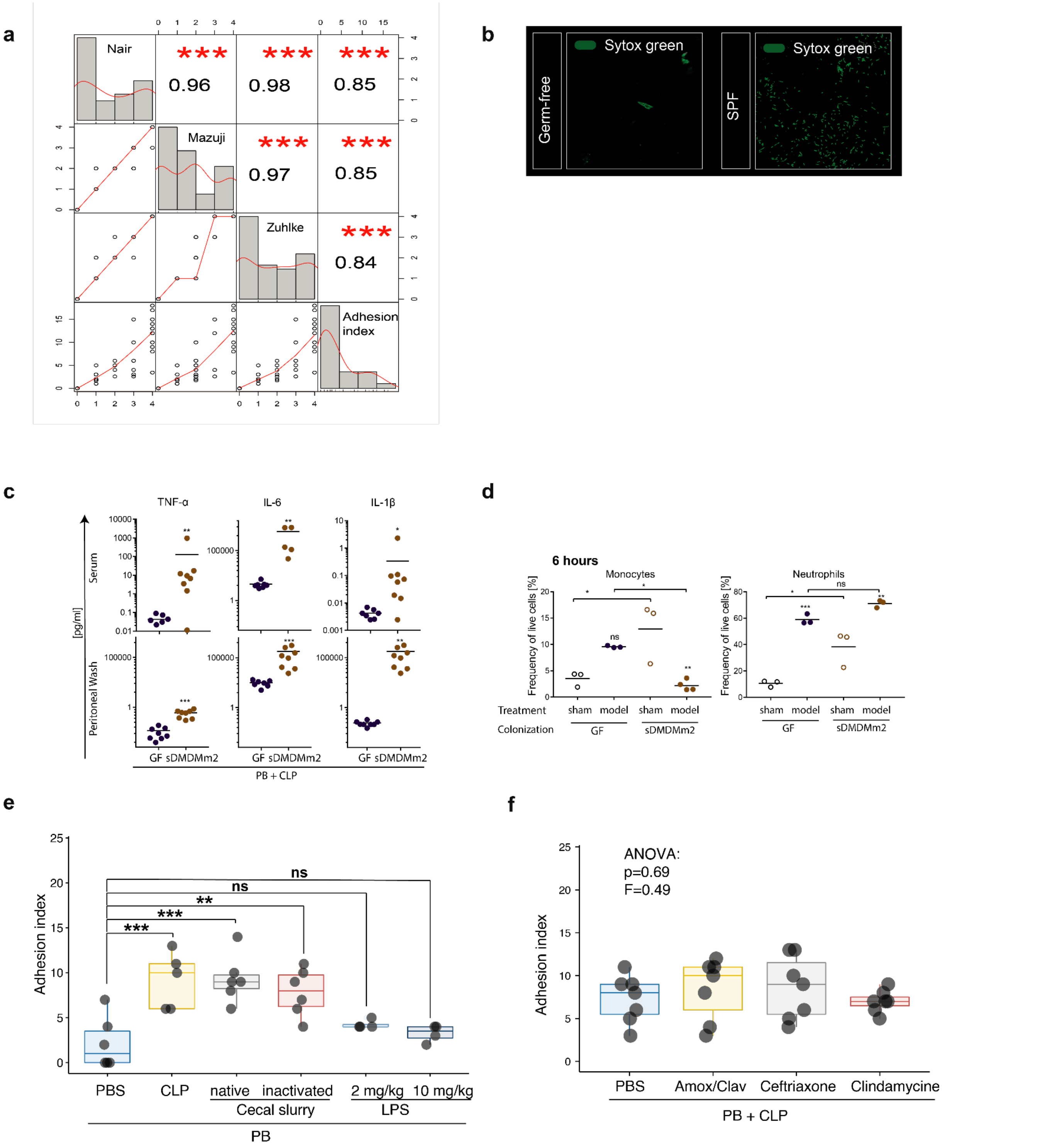

Supplemental Figure 1. Validation of mouse model. a Correlation matrix comparing adhesion index to published Nair, Mazuji and Zuhlke adhesion scores. **b** Sytox green staining of feces samples of mice 7 days post-surgery shows that germ-free animals remained germ-free. **c** Cytokines (tumor necrosis factor alpha (TNF-alpha), interleukin 6 (IL-6), interleukin 1 beta (IL-1 beta)) in peritoneal cavity fluid and serum of germ-free (GF) and gnotobiotic (sDMDMm2) mice collected 7 days after mice underwent peritoneal buttons and cecal ligation and puncture (PB + CLP). Data represent n=8 independent animals per group

examined over one independent experiment. Data are presented as mean and individual values. Significance by Mann-Whitney tests (two-tailed) without correction for multiple testing. Serum TNF-alpha: p=0.0093, Serum IL-6: p=0.0025, Serum IL-1 beta: p=0.010, Peritoneal TNF-alpha: p=0.0002, Peritoneal IL-6: p=0.0003, Peritoneal IL-1 beta: p=0.0028, **d** Cell counts (flow cytometry) in the peritoneal fluid 6 hours post-surgery. Data represent n=3 for GF sham, 3 for GF model, 3 for sDMDMm2 sham, and 4 for sDMDMm2 model independent animals examined over one independent experiment. Data are presented as mean and individual values. P-values by one-way ANOVA with Tukey's post hoc test. Exact p-values in Supplementary Data 1. **e** Adhesion index 7 days post-surgery. All mice received peritoneal buttons (PB), in addition mice received either phosphate buffered saline (n=6), cecal ligation and puncture (CLP, n=5), cecal slurry (CS native, n=6), heat inactivated cecal slurry (n=6), or lipopolysaccharide (LPS) in two different doses (n=4 each). Data represent independent animals examined and pooled from three independent experiments. Data are presented as individual values and boxplots (median, first and third quartile). Adjusted p-values by one-way ANOVA with Tukey's post hoc test. PBS vs. CLP: p= 0.001, PBS vs. native cecal slurry: p= 0.00047, PBS vs. heat inactivated cecal slurry: p= 0.0064, PBS vs. 2mg/kg LPS: p=0.78, PBS vs. 10mg/kg LPS: p=0.98. **f** Adhesion index 7 days post-surgery. All mice received PB + CLP. In addition, mice received one of three broad spectrum antibiotics 30-60 min prior to surgery, or the respective phosphate buffered saline (PBS) control. Data represent n=7 independent animals per group, examined in one experiment. Data are presented as individual values and boxplots (median, first and third quartile). P-value and F-value by one-way ANOVA. *P<0.05, **P<0.01, ***P<0.001, ****P<0.0001, n.s. P≥0.05. PBS: phosphate buffered saline.

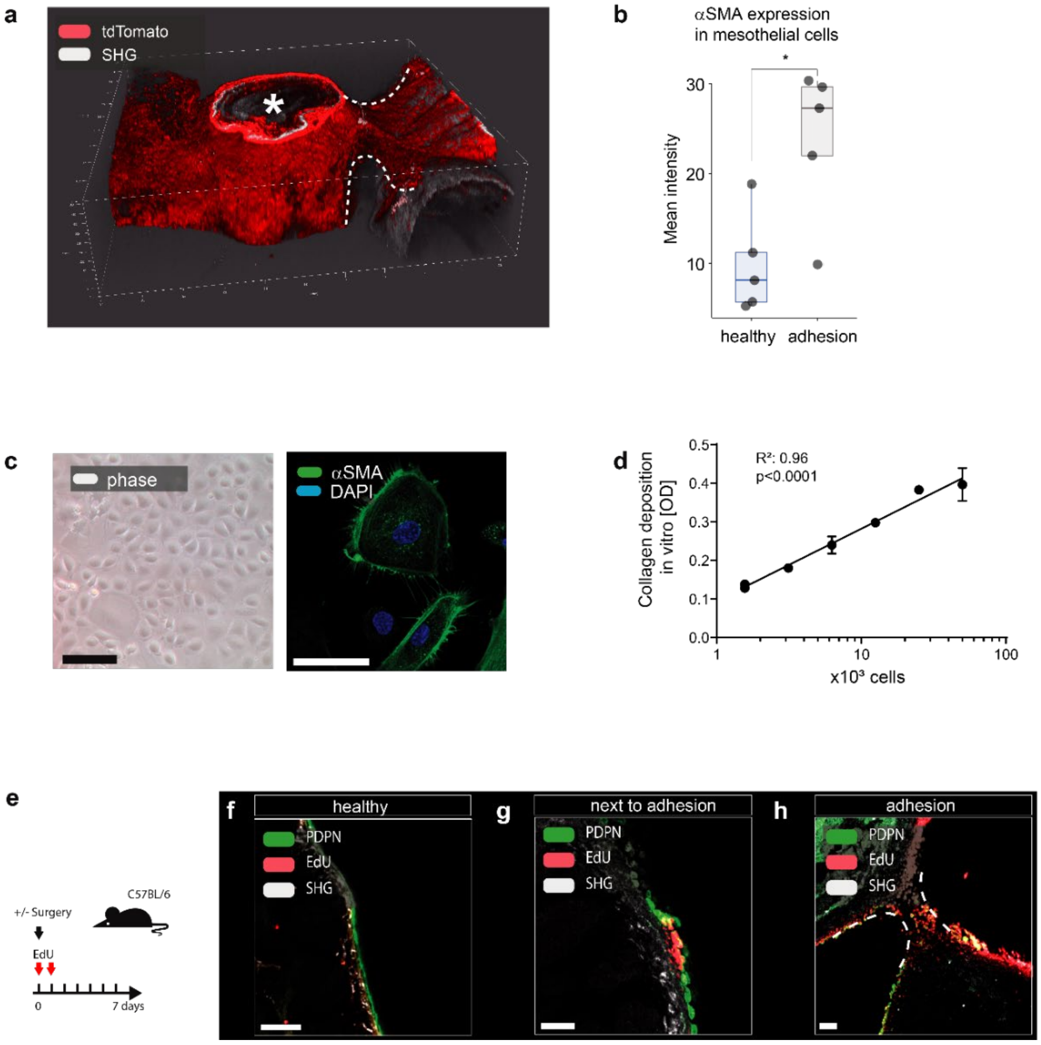

Supplemental Figure 2. Increase in post-surgical collagen deposition is correlated with activation and proliferative expansion of mesothelial cells. a Three-dimensional reconstruction of an adhesion biopsy of a *Wt1*^{CreERT2} *Rosa26*^{tdTomato} reporter mouse obtained 7 days post-surgery. Tissue was cleared and optically sectioned. Collagen is indicated by its second harmonic generation (SHG) Scale bar left panel: 250 µm, right panel: 50 µm. **b** Alpha smooth muscle actin expression (αSMA, mean fluorescent intensity) in cells of mesothelial origin (*Wt1*^{CreERT2} *Rosa26*^{tdTomato} positive) was measured in healthy regions and adhesion biopsies 7 days after surgery. P=0.032. **c** Mesothelial cells in culture for 7 days show cobble-stone pattern and start to express αSMA. Scale bar left panel: 250µm. Scale bar right panel: 50µm. **d** Amount of collagen secretion in vitro (5 days) correlates with number of mesothelial cells seeded. **e** 5-ethynyl-2-deoxyuridin (EdU) was administered twice in the combined injury + CLP model as well as in unoperated control animals during the first twenty-four hours post-surgery. Data represent three technical replicates of n=1 biological sample representative of n=2 independent experiments. Data represent mean +/- standard deviation. **f-h** Whole mount immunohistochemistry stained for mesothelium marker podoplanin (PDPN) and EdU incorporation. Biopsies were obtained from healthy abdominal wall (f), abdominal wall next to adhesions (g) and adhesions themselves (h). Scale bar: 50 µm. Data are represented as median +/- interquartile range (each symbol represents 1 mouse) (b) n=5 per group, representative of three independent experiments. (e-h) n=3 per group, representative of two independent experiments. P-values by Wilcoxon test (two-sided) with Holm-Bonferroni correction for multiple-testing. *P<0.05

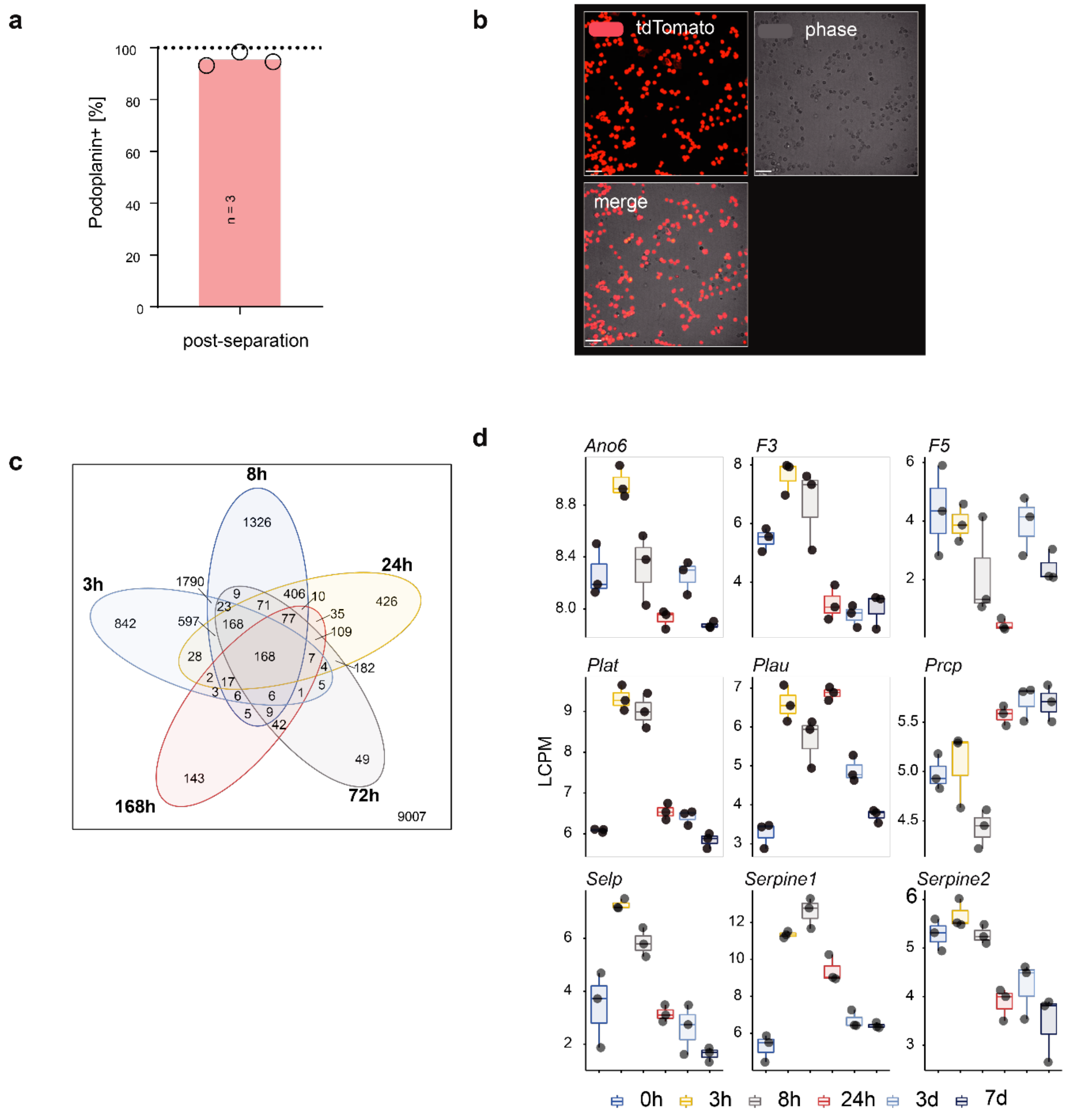

Supplemental Figure 3. Mesothelial cells undergo a profound transcriptional change post-surgery. a Cells isolated using anti-glycoprotein M6A (GPM6A) magnetic beads were counterstained with podoplanin to validate purity. Data are presented as individual values and mean. **b** Cytospin of cells isolated with anti-GPM6A magnetic beads in *Wt1*^{CreERT2} *Rosa26*^{tdTomato} reporter mice that were treated with Tamoxifen. Image is representative of n=3 independent animals of one independent experiment. Scale bar: 50µm. **c** Venn diagram with numbers of genes differentially expressed versus baseline. **d** Mesothelial expression (log count per million, LCPM) of genes associated with coagulation. Data represent n=3 independent animals of one independent experiment. Data are presented as individual values and boxplots (median, first and third quartile).

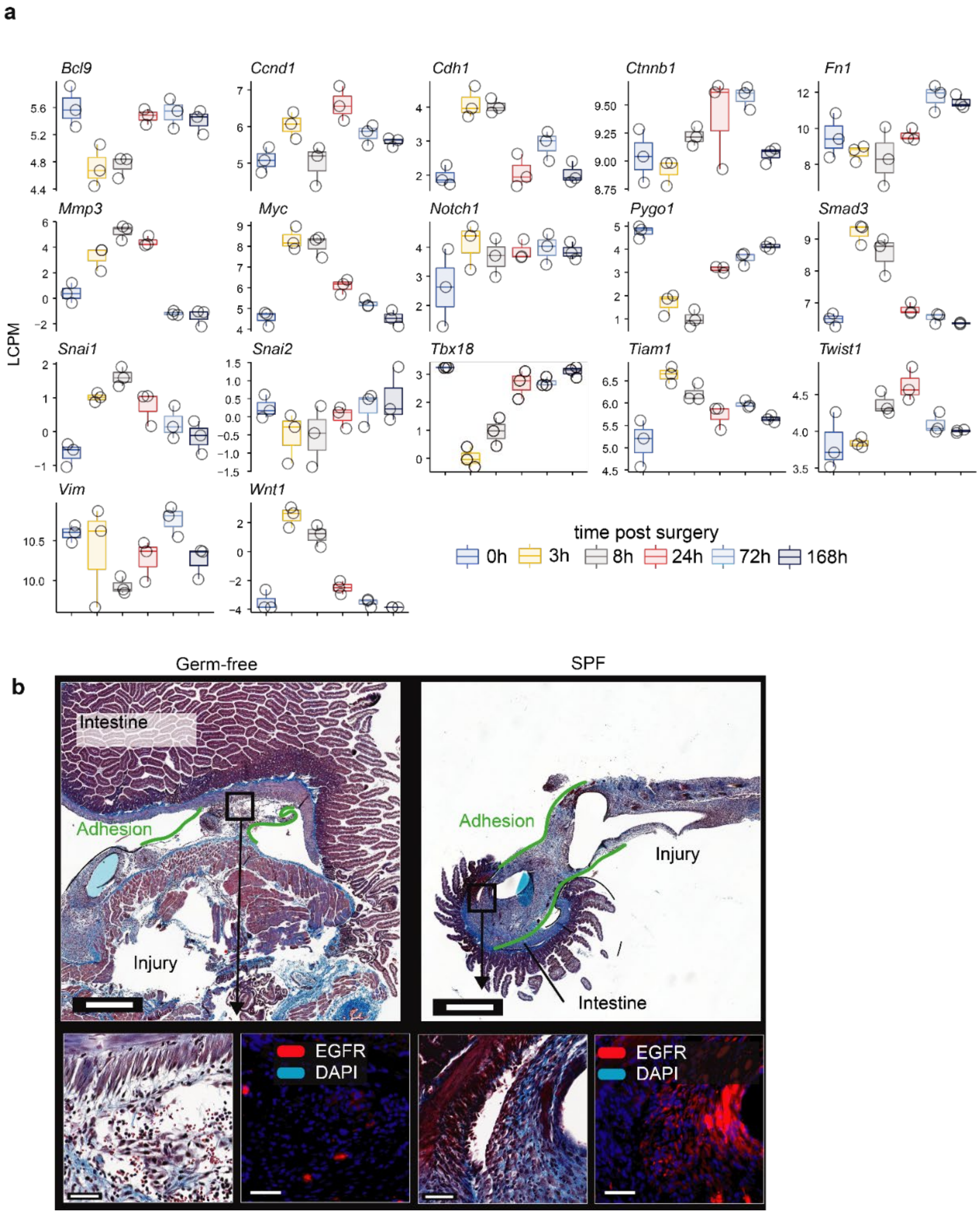

Supplemental Figure 4. Mesothelial cell activation is driven by receptor tyrosine kinases of the ERBB family. **a** Mesothelial expression (log count per million, LCPM) of genes associated with mesothelial to mesenchymal transition. Data represent n=3 independent animals of one independent experiment. Data are presented as individual values and boxplots (median, first and third quartile). **b** Adhesion biopsies from germ-free (GF) and specific-pathogen-free (SPF) mice, 7 days post-surgery (PB + CLP). Masson trichrome staining and Immunohistochemistry for epidermal growth factor receptor (EGFR). Scale bar overview: 500 µm, Scale bar magnification: 50 µm. The images are representative of n=8 (GF) and n=7 (SPF) animals examined over two independent experiments.

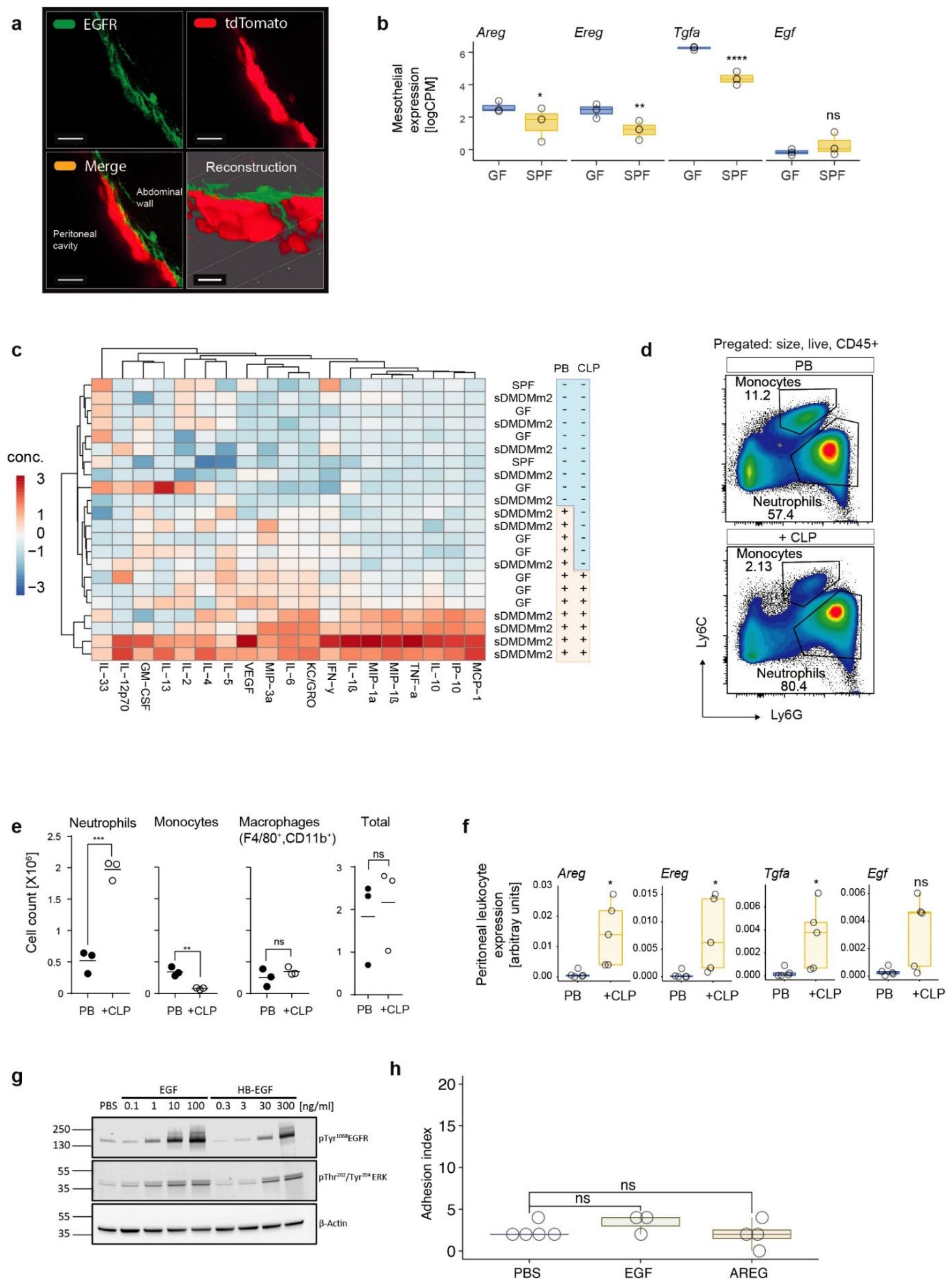

Supplemental Figure 5. Peritoneal fluid cytokine profile. a Whole mount immunohistochemistry of healthy abdominal wall biopsies from *Wt1*^{CreERT2} *Rosa26*^{tdTomato} reporter mice. Epidermal growth factor receptor (EGFR) at baseline is predominantly expressed on the basolateral side. Scale bar: 10μm. **b** Mesothelial cell EGFR-ligand expression (RNA-Seq) of germ-free (GF)

and specific-pathogen-free (SPF) mice 3 hours after surgery (amphiregulin (*Areg*), epiregulin (*Ereg*), transforming growth factor alpha (*Tgfa*), epidermal growth factor (*Egf*)). Data represent n=3 independent animals of one independent experiment. Data are presented as individual values and boxplots (median, first and third quartile). P-values by linear regression modeling described in methods section. Exact p-values in Supplementary Data 1. **c** Peritoneal washes from specific-pathogen-free (SPF), gnotobiotic (stable defined moderately diverse mouse microbiota, sDMDMm2) and germ-free (GF) mice that were either untreated or 7 days after sterile injury (PB) alone or in combination with bacterial contamination (+CLP). Multiplexed mesoscale cytokine/chemokine assay was performed. Hierarchical clustering of the results. Each horizonal line represents one mouse. N=3-7 per group. **d** Flow cytometry results of peritoneal wash 7 days after sterile injury alone (PB) or in combination with bacterial contamination (+CLP). Cells are single cells, gated on live staining^{-}, CD45^{+} and CD19^{-}. **e** Cell counts by flow cytometry of peritoneal wash 7 days after sterile injury alone (PB) or in combination with bacterial contamination (+CLP). Data represent n=3 independent animals of one independent experiment. Data are presented as mean + individual values. P-values by unpaired t-test (two-tailed) without correction for multiple testing. Neutrophils: p=0.0005, Monocytes: p=0.0034, Macrophages: p=0.35, Total: p=0.70. **f** EGFR-ligand expression of leukocytes isolated by peritoneal wash from SPF mice 24 hours after sterile injury alone (PB) or in combination with bacterial contamination (+CLP). Data represent n=5 independent animals of one independent experiment. Data are presented as individual values and boxplots (median, first and third quartile). P-values by Wilcoxon test (two-sided) without correction for multiple testing. Areg: p=0.0079, Ereg:p=0.0079, Tgfa: p=0.032, Egf; p=0.095. **g** Western blot stained for phospho-EGFR and phospho-ERK. Primary mesothelial cells were incubated with epidermal growth factor (EGF) and heparin-binding epidermal growth factor (HB-EGF) with the indicated doses for 20 minutes. **h** Adhesion index 7 days post-surgery. All mice received PB only. In addition, mice were injected intraperitoneally with either phosphate buffered saline (PBS) or 5µg of either epidermal growth factor (EGF) or amphiregulin (AREG) daily. P-values by Wilcoxon test (two-sided) with Holm-Bonferroni correction for multiple-testing. PBS vs. EGF: p=0.29, PBS vs. AREG: p=0.77. Data represent n=5,3 and 4 independent animals of one independent experiment. Data are presented as individual values and boxplots (median, first and third quartile). *P<0.05, ***P<0.001, n.s. P≥0.05.

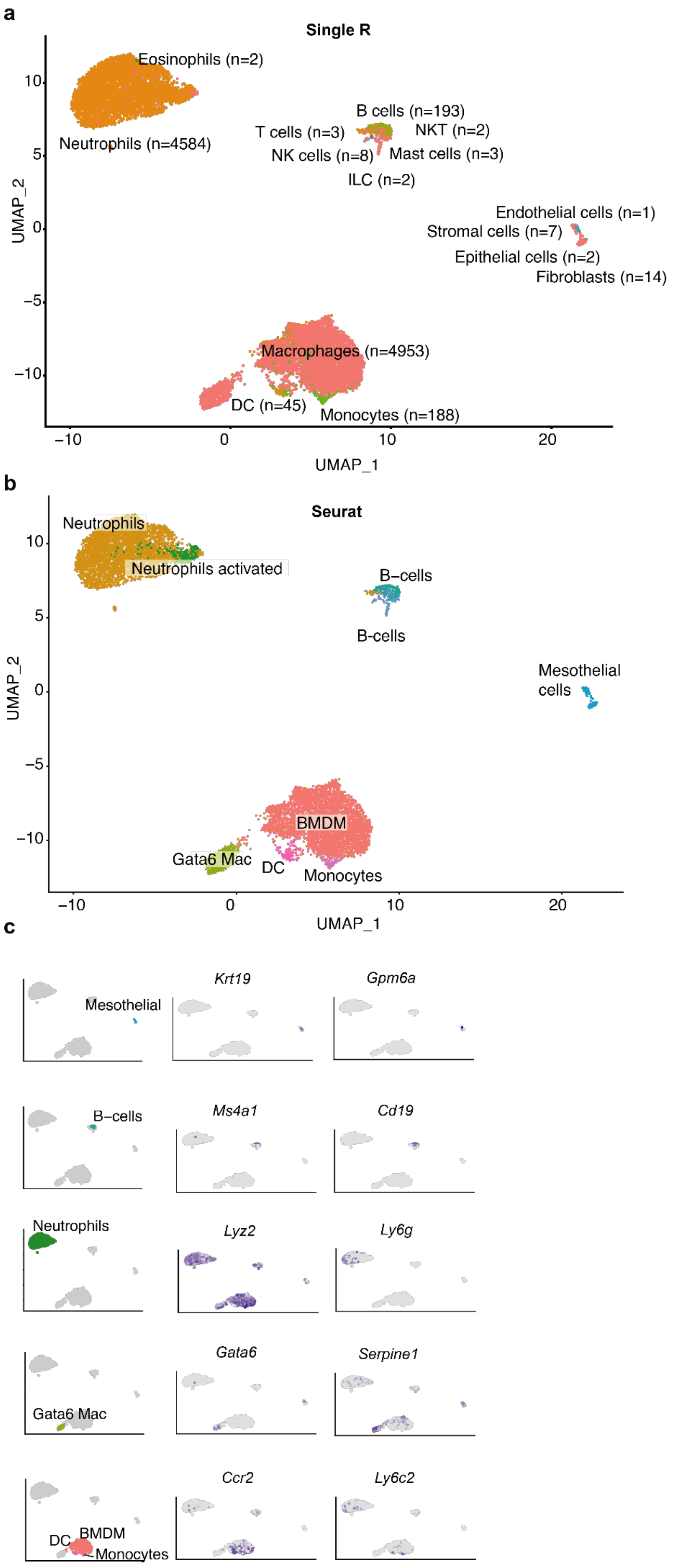

Supplemental Figure 6. Manual cluster annotation vs SingleR. a-c UMAP plots with cells colored according to Seurat clusters are shown. The manually merged and annotated cluster names (b) which are based on characteristic expression markers (c) were validated against an automated cluster annotation (SingleR package) (a) which is completely user independent.

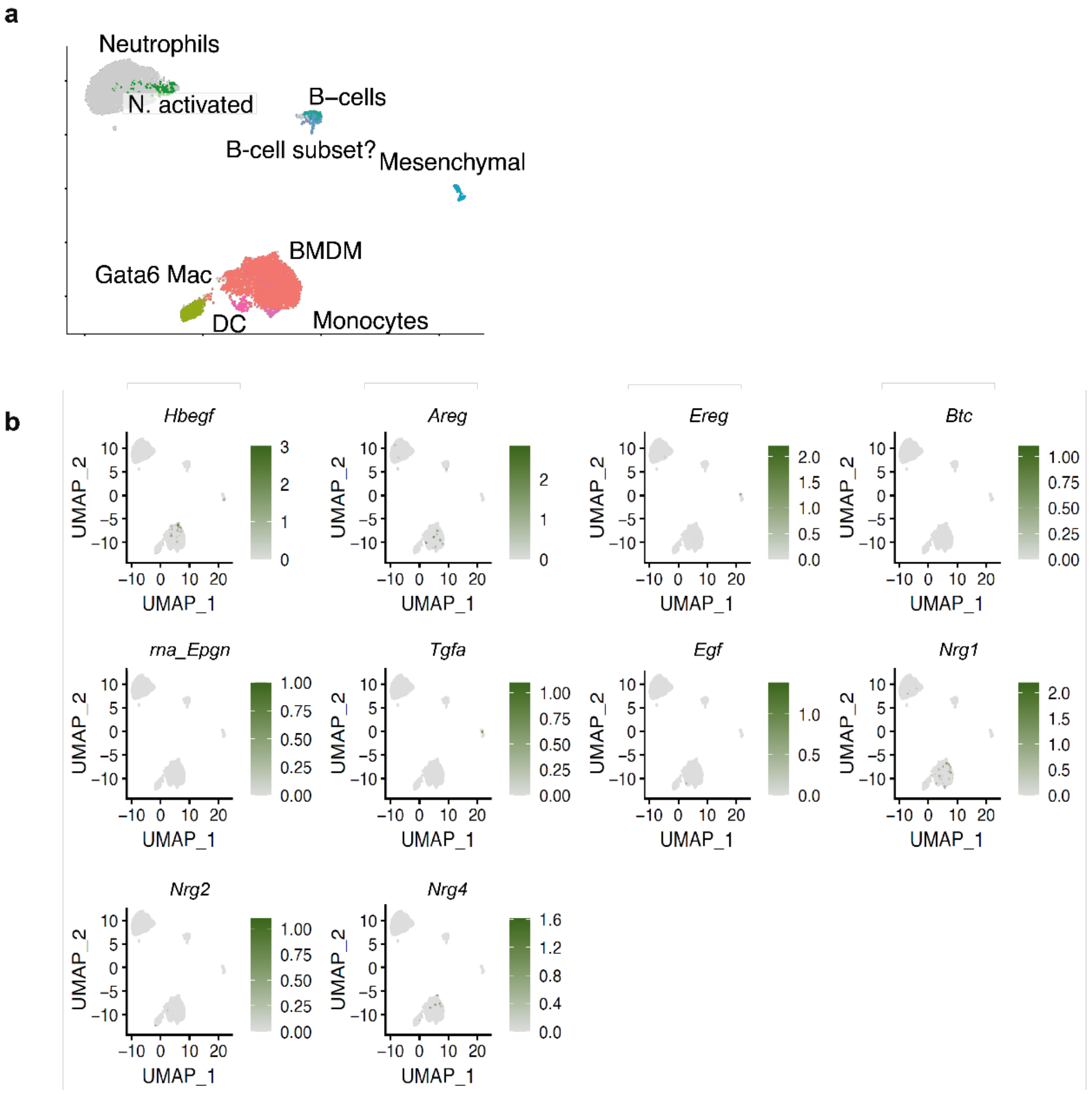

Supplemental Figure 7. Expression of all known EGFR ligands. a-b UMAP plots with cells colored according to Seurat clusters (a) and scRNA-Seq expression levels for all known ligands for epidermal growth factor receptor (EGFR) (b) are shown.

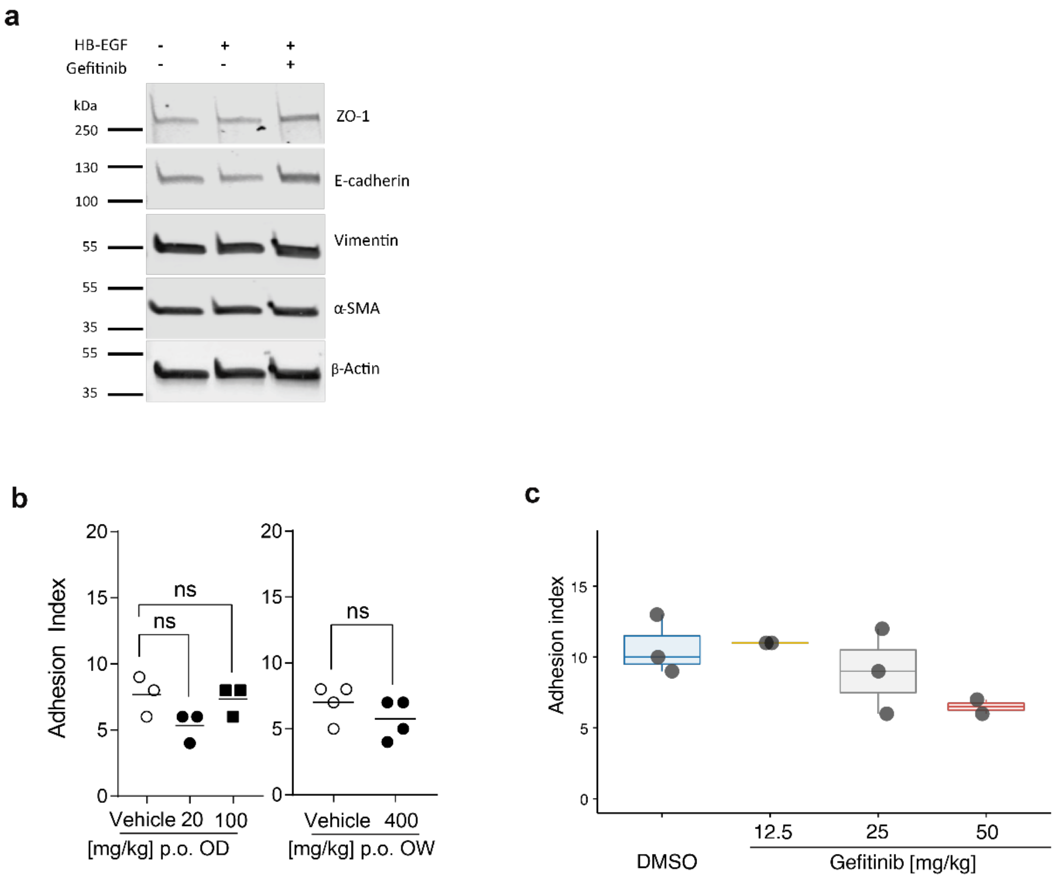

Supplemental Figure 8. Effects of Gefitinib in vitro and in vivo. a Western blot of protein lysate from primary mesothelial cells treated with heparin-binding epidermal growth factor (HB-EGF, 300ng/ml) and small molecule inhibitor Gefitinib (500nM). Protein expression of zonula occludens protein 1 (ZO-1), E-cadherin, vimentin, and alpha smooth muscle actin (α-SMA) is displayed. **b** Adhesion index 7 days after surgery (PB + CLP). DMSO: Dimethylsulfoxide. OD: once daily, OW: once weekly. Data represent n=3 (left panel) or n=4 (right panel) independent animals of one independent experiment. Data are presented as mean + individual values. Vehicle vs. 20: p=0.2, Vehicle vs. 100: p=0.99, Vehicle vs. 400: p=0.31. **c** Adhesion index 7 days after surgery (PB + CLP). Data represent n=3 individual animals per group (except 12.5mg/kg group only n=2). Data are presented as individual values + boxplot (median, first and third quartile). P-values by Wilcoxon test (two-sided) with Holm-Bonferroni correction for multiple-testing, n.s. P≥0.05.

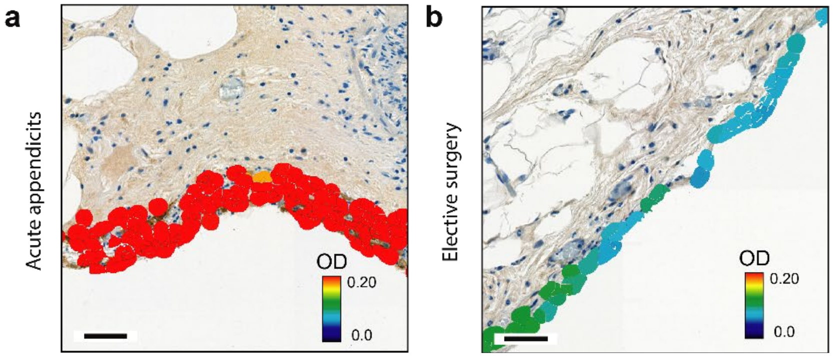

Supplemental Figure 9. QuPath quantification of mesothelial EGFR expression. a and b Biopsies from patients that underwent surgery due to acute appendicitis (a) or for elective abdominal surgery such as tumor surgery (b). Stained with anti-human epidermal growth factor receptor (EGFR). Automated cell segmentation of mesothelium using QuPath software. Color scale indicates mesothelial EGFR-expression. OD: optical density scale. Scale bar: 200um. The images are representative of the quantification results shown in Fig. 7e-f and the numbers and demographics outlined in Supplementary Table 1.

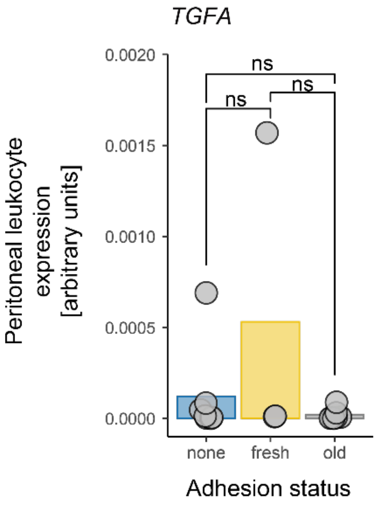

Supplemental Figure 10. Human peritoneal leukocyte TGF alpha expression. Peritoneal leukocytes were isolated from patients with no, fresh, or old adhesions. Expression of transforming growth factor alpha (*TGFA)* was measured by quantitative polymerase chain reaction. Data represent n=8 for none, 4 for fresh, and 9 for old individual patients of one independent cohort study. Patient demographics according to Supplementary Table 2. Data are represented as mean and individual symbols. P-values by Wilcoxon test (two-sided) with Holm-Bonferroni correction for multiple-testing, None vs. fresh: p=0.67, none vs. old: p=0.53, fresh vs. old: p=0.18. n.s. P≥0.05.

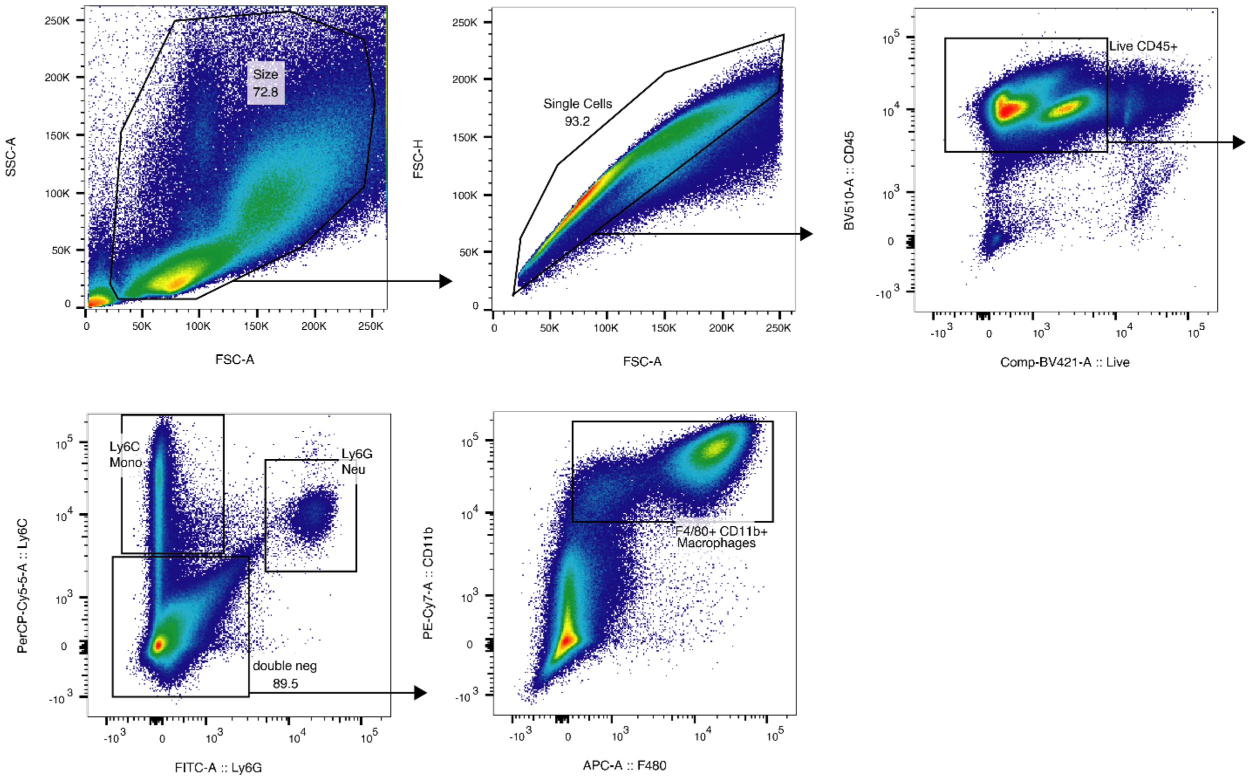

Supplemental Figure 11. Flow cytometry gating strategies. Representative example of gating strategy used for data shown in Fig. S1d and Fig. S5d,e.

Supplementary Table 1. **Patient demographics appendicitis cohort**

	Appendicitis (N=11)	Elective (N=7)	Overall (N=18)
**Gender**			
female	4 (36.4%)	3 (42.9%)	7 (38.9%)
male	7 (63.6%)	4 (57.1%)	11 (61.1%)
**Age**			
Mean (SD)	39.7 (24.6)	66.3 (12.1)	50.1 (24.2)

Supplementary Table 2. **Patient demographics adhesion cohort**

	Adhesion status<br>none<br>(N=8)	fresh<br>(N=4)	old<br>(N=9)	Total<br>(N=21)
**Gender**				
female	5 (62.5%)	2 (50.0%)	3 (33.3%)	10 (47.6%)
male	3 (37.5%)	2 (50.0%)	6 (66.7%)	11 (52.4%)
**Age**				
Mean (SD)	54.9 (20.2)	59.3 (16.2)	66.4 (17.2)	60.7 (18.1)

Supplementary Table 3. **Antibodies for Histology**

**Antibody**	**Company (catalog no.)**	**Fixation**	**Dilution**
**Primary**			
Rat anti-mouse M6A	MBL (D055-3)	Cryo	1:100
Anti-human mesothelin	R&D (MAB32651)	PFA	1:25
Anti-human EGFR	(Dako-Agilent, M7239, Clone E30)	PFA	1:25
Anti-human Calretinin	Leica Biosystems (NCL-L-CALRET-566)	PFA	1:100
Anti-human Cytokeratin	Agilent (M3515)	PFA	1:100
**Secondary**			
Alexa Fluor 546 goat anti-rat IgG	Invitrogen (A-11081)		1:200
Biotinylated goat anti-rat	DAKO (E0466)	PFA	1:200
Biotinylated goat anti-rabbit	Vector laboratories (BA-1000)	PFA	1:200
**Conjugated**			
Alexa Fluor 488 anti-mouse podoplanin	eBioscience (53-5381-82)	In vivo injection before tissue fixation	5ug intra peritoneal injection
Alexa Fluor 488 anti-mouse alpha smooth muscle actin	eBioscience (53-9760-82)	PFA	1:200

Supplementary Table 4. Primers

Gene	Primer 1	Primer 2	Company (catalog no.)
**Mouse**			
Areg	*5'-GTCACTATCTTTGTCTCTGCCA-3'*	*5'-CCTCCTTCTTTCTTCTGTTTCTCC-3'*	Integrated DNA technologies (208924660)
Ereg	*5'-CTTCTACAGGCAGTTATCAGCA-3'*	*5'-GTAGCCGTCCATGTCAGAAC*	Integrated DNA technologies (208924663))
Tgfa	*5'-GCTGTCCTCATTATCACCTGT-3'*	*5'-GCAAGCAGTCCTTCCCTT-3'*	
Egf	*5'-GTCCTAGAGAAACACCAAGACC-3'*	*5'-TCCATGAAGTCAGATGCACTG-3'*	Integrated DNA technologies (208924669)
**Human**			
AREG	Thermo Fisher Scientific	Hs00950669_m1*	4331182
EREG	Thermo Fisher Scientific	Hs00914313_m1*	4331182
TGFA	Thermo Fisher Scientific	Hs00177401_m1*	4331182
EGF	Thermo Fisher Scientific	Hs01099990_m1	4331182

*The sequences of the human primers are propieraty (Thermo Fisher Scientific, TaqMan®)

Supplementary Table 5. Antibodies for flow cytometry

Antibody	Clone (catalog no.)	Dye	Dilution
Rat Anti-Mouse CD45	30-F11 (BD, #564279)	BUV 395	1:200
Rat Anti-Mouse Ly6G	RB6-8C5 (Invitrogen, #25-5931-81)	PE-Cy7	1:200
Rat Anti-Mouse Ly6C	HK1.4 (Invitrogen, #45-5932-80)	PerCP-Cy5.5	1:200
Anti-mouse podoplanin	eBioscience (53-5381-82)	Alexa Fluor 488	1:200

Supplementary Table 6. Antibodies for Western Blot

Antibody	Clone (catalog no.)	Dilution
Primary		
Anti-mouse EGFR	D38B1 (Cell signaling #4267)	1:1000
Anti-mouse p-EGFR (Tyr1068)	D7A5 (Cell signaling #3777)	1:1000
Anti-mouse Akt	Polyclonal (Cell signaling #9272)	1:1000
Anti-mouse p-Akt (Thr308)	D25E6 (Cell signaling #13038)	1:1000
Anti-mouse Stat3	124H6 (Cell signaling #9139)	1:1000
Anti-mouse p-Stat3 (Ser727)	Polyclonal (Cell signaling #9134)	1:1000
Anti-mouse p44/42 MAPK (Erk1/2)	Polyclonal (Cell signaling #9102)	1:1000
Anti-mouse p-p44/42 MAPK (Erk1/2) (Thr202/Tyr204)	Polyclonal (Cell signaling #9101)	1:1000
Anti-mouse α-SMA	1A4 (Sigma-Aldrich #A5228)	1:1000
Anti-mouse Vimentin	EPR3776 (abcam #ab92547)	1:1000
Anti-mouse E-cadherin	36/E-Cadherin (BD Bioscience #610181)	1:1000
Secondary		
Goat anti-Mouse IgG IRDye® 800CW	Polyclonal (Licor #925-32210)	1:1000
Goat anti-Rabbit IgG IRDye® 680LT	Polyclonal (Licor #925-68021)	1:1000
Goat anti-Rabbit IgG IRDye® 800CW	Polyclonal (Licor #925-32211)	1:1000
Goat anti-Rat IgG IRDye® 680LT	Polyclonal (Licor #925-68029)	1:1000
Conjugated		
HRP-conjugated β-actin	AC-15 (Sigma-Aldrich #A3854)	1:50000
