# Supplementary material for: Intraperitoneal microbial contamination drives post-surgical peritoneal adhesions by mesothelial EGFR-signaling
Source: Nat Commun. 2021 Dec 16;12:7316. doi: 10.1038/s41467-021-27612-x (PMC8677808; doi:10.1038/s41467-021-27612-x)
Supplement: Supplementary file 3 — Description of Additional Supplementary Files [file 41467_2021_27612_MOESM3_ESM.pdf]

Description of Additional Supplementary Files

File Name: **Supplementary Data 1**
Description: **Worksheet 1-2.** The output of the differential gene expression analysis by linear modelling with limma (described in methods section) is presented as log-fold change (logFC), moderated t-statistic (t) and adjusted p-value (P-value) for each gene. For each gene ENSEMBL ID, gene symbol and the gene biotype (e.g. protein coding) is provided. Differences of logFC > 2 or logFC < -2 with p<0.05 were considered significantly up (+1) or down (-1) regulated (updown) respectively, all other genes were considered unchanged (0). The applied contrasts (comparisons) were 3, 8, 24, 72 and 168 hours versus 0 hours for (worksheet 1) **which is relevant for Figures 4a-b.** The contrasts for (worksheet 2) which are **relevant for Figures 4c and S5b** were germ free (GF) versus specific pathogen free (SPF) at 0, 3,24 and 72 hours respectively. **Worksheet 3.** Statistics including the exact P-values generated by a Tukey's multiple comparison test of the data shown in Supplementary Figure 1d.
