# Supplementary material for: Intraperitoneal microbial contamination drives post-surgical peritoneal adhesions by mesothelial EGFR-signaling
Source: Nat Commun. 2021 Dec 16;12:7316. doi: 10.1038/s41467-021-27612-x (PMC8677808; doi:10.1038/s41467-021-27612-x)
Supplement: Supplementary file 5 — Reporting Summary [file 41467_2021_27612_MOESM5_ESM.pdf]

nature research

Corresponding author(s): Joel Zindel

Last updated by author(s): Nov 8, 2021

Reporting Summary

Nature Research wishes to improve the reproducibility of the work that we publish. This form provides structure for consistency and transparency in reporting. For further information on Nature Research policies, see our Editorial Policies and the Editorial Policy Checklist.

Statistics

For all statistical analyses, confirm that the following items are present in the figure legend, table legend, main text, or Methods section.

n/a	Confirmed	
☐	☒	The exact sample size (*n*) for each experimental group/condition, given as a discrete number and unit of measurement
☐	☒	A statement on whether measurements were taken from distinct samples or whether the same sample was measured repeatedly
☐	☒	The statistical test(s) used AND whether they are one- or two-sided<br>*Only common tests should be described solely by name; describe more complex techniques in the Methods section.*
☒	☐	A description of all covariates tested
☒	☐	A description of any assumptions or corrections, such as tests of normality and adjustment for multiple comparisons
☐	☒	A full description of the statistical parameters including central tendency (e.g. means) or other basic estimates (e.g. regression coefficient) AND variation (e.g. standard deviation) or associated estimates of uncertainty (e.g. confidence intervals)
☐	☒	For null hypothesis testing, the test statistic (e.g. *F*, *t*, *r*) with confidence intervals, effect sizes, degrees of freedom and *P* value noted<br>*Give P values as exact values whenever suitable.*
☒	☐	For Bayesian analysis, information on the choice of priors and Markov chain Monte Carlo settings
☒	☐	For hierarchical and complex designs, identification of the appropriate level for tests and full reporting of outcomes
☒	☐	Estimates of effect sizes (e.g. Cohen's *d*, Pearson's *r*), indicating how they were calculated

Our web collection on statistics for biologists contains articles on many of the points above.

Software and code

Policy information about availability of computer code

Data collection	Flow cytometry data was collected using BD FACSDiva software (version 8)and exported as FCS3 files. Whole mount microscopy images were acquired using a Leica SP8 2-photon microscope in inverted confirguration operated by Leica LAS X (v. 3.7) software. Slides were scanned using Panoramic scanner software from (3DHISTECH, version 3.0.2). Real-time PCR was performed using ABI 7900 with SDS 2.3 software. Mesoscale plates were measured suing the Meso Scale Discovery system (MSD, Rockvielle, Maryland). RNA-Seq and scRNA-Seq data collection was done using a Nova Seq 6000 operated by NovaSeq Control Software (v. 1.5 for bulk RNA-Seq, v. 1.7 for scRNA-Seq). Immunoblot images were acquired using Li-cor Odyssey infra-red scanner operated by with Li-cor Odyssey software (2.1.15) or FusionCapt Advance FX7 operated by the latest firmware (version 1.0.12). Human data were collected using REDCap (v 11.0.3) browser based software (cohort 2) or Excel spread sheets (cohort 2) secured on an institutional server.
Data analysis	Image analysis was done using QuPath (v.0.1.2) and ImageJ (v. 2.0.0, Java 1.8.0) open source software. Flow cytometry fcs files were analysed with Flowjo (Treestar). Fastq files were generated by Illumina sequencer software. Alignment was performed using hisat (2.1.0) for bulk RNA-Seq data and Cell Ranger (6.1.1) for scRNA-Seq data using the reference genome mm10. For bulk RNA-Seq the features were counted using the FeatureCount function of the package Rsubred (3.11). The collected and pre-processed data was exported for analysis on a personal computer using R:<br>- R version 4.0.3 (2020-10-10), Platform: x86_64-w64-mingw32/x64 (64-bit), Running under: Windows 10 x64 (build 19042)<br>- Packages<br>--Bulk RNA Seq: Mus.musculus_1.3.1, limma_3.46.0, Glimma_2.0.0, biomaRt_2.46, edgeR_3.32.1<br>--scRNA-Seq: CellRanger (6.1.1), R packages: DoubletFinder_2.0.3, Seurat_4.0.1, SingleR_1.4.1, Rmagic_2.0.3<br>--other packages used in this project: ggpubr_0.4.0, ggpmisc_0.3.8-1, ggsci_2.9, gridExtra_2.3, forcats_0.5.1, dplyr_1.0.5, purrr_0.3.4, readr_1.4.0, tidyr_1.1.3, tibble_3.1.0, ggplot2_3.3.3, tidyverse_1.3.0, scales_1.1.1, stringr_1.4.0, readxl_1.3.1, data.table_1.14.0, pspline_1.0-18, viridis_0.5.1, viridisLite_0.3.0, ggpubr_0.4.0 , gtools_3.8.2, TxDb.Mmusculus.UCSC.mm10.knownGene_3.10.0 org.Mm.eg.db_3.12.0.<br>Differentially expressed genes by limma pairwise comparison was subjected to gene set enrichment analysis using metascape (http://

metascape.org) using the latest version of the database at the time of analysis (last updated on 2017-03-16).

For manuscripts utilizing custom algorithms or software that are central to the research but not yet described in published literature, software must be made available to editors and reviewers. We strongly encourage code deposition in a community repository (e.g. GitHub). See the Nature Research guidelines for submitting code & software for further information.

Data

Policy information about availability of data

All manuscripts must include a data availability statement. This statement should provide the following information, where applicable:

- Accession codes, unique identifiers, or web links for publicly available datasets
- A list of figures that have associated raw data
- A description of any restrictions on data availability

(RNA-Seq data and scRNA-Seq data are publicly available (GEO). The RNA-Seq data generated in this study have been deposited in the Genome Expression Omnibus (GEO) database under accession code GSE156127 [https://www.ncbi.nlm.nih.gov/geo/query/acc.cgi?acc=GSE156127]. The scRNA-Seq data are deposited in the GEO database under accession code GSE186658 [https://www.ncbi.nlm.nih.gov/geo/query/acc.cgi?acc=GSE186658]. The remaining data are available within the Article, Supplementary Information or Source Data file.

Figures with associated raw data:
- Fig 1d, e, f
- Fig 2d, i
- Fig 5g
- Fig 6b, c, e, f, g, i
- Fig 7e-g
- Fig 8b-d

Publicly available databases used in this study:
- mm10 genome assembly (https://www.ncbi.nlm.nih.gov/assembly/GCF_000001635.20/)

Field-specific reporting

Please select the one below that is the best fit for your research. If you are not sure, read the appropriate sections before making your selection.

☒ Life sciences ☐ Behavioural & social sciences ☐ Ecological, evolutionary & environmental sciences

For a reference copy of the document with all sections, see nature.com/documents/nr-reporting-summary-flat.pdf

Life sciences study design

All studies must disclose on these points even when the disclosure is negative.

Sample size	For the exploratory experiments of this project not enough prior data was available to perform formal sample size calculations. The sample size was generally chosen to detect an expected effect size >1.5 or higher with a power of 80%. This approach was sufficient to detect pronounced biological effects with high potential for translational value.
Data exclusions	As per protocol the animals were excluded due to loss of follow up due to death (e.g. anesthesia) or euthanasia (e.g. weight loss > 20%, pain score above threshold).
Replication	Experiments were replicated independently as indicated in the figure legends. The data shown in the figures are representative of all replications or comprise a pooled analysis of the replications. Pooling is explicitly stated in the figure legends.
Randomization	Mice were randomly allocated to different treatments by random number generation. Randomization was stratified by cage and genotype ensuring that co-variates such as age or cage- and batch-specific unknown covariates were controlled.
Blinding	Investigators were blinded during treatment and adhesion scoring of mice treated pharmacologically. Different surgical models were blinded for adhesion scoring where feasible. Comparison of different hygiene status (e.g. germ free vs. colonized mice) was blinded for adhesion scoring. The investigators were blinded to group allocation during data analysis for all experiments.

Reporting for specific materials, systems and methods

We require information from authors about some types of materials, experimental systems and methods used in many studies. Here, indicate whether each material, system or method listed is relevant to your study. If you are not sure if a list item applies to your research, read the appropriate section before selecting a response.

Materials & experimental systems

n/a	Involved in the study
☐	☒ Antibodies
☒	☐ Eukaryotic cell lines
☒	☐ Palaeontology and archaeology
☐	☒ Animals and other organisms
☐	☒ Human research participants
☒	☐ Clinical data
☒	☐ Dual use research of concern

Methods

n/a	Involved in the study
☒	☐ ChIP-seq
☐	☒ Flow cytometry
☒	☐ MRI-based neuroimaging

Antibodies

Antibodies used

Supplemental Table 3. Antibodies for Histology

Primary
Rat anti-mouse M6A MBL (D055-3) Ccryo 1:100
Anti-human mesothelin R&D (MAB32651) PFA 1:25
Anti-human EGFR (Dako-Agilent, M7239, Clone E30) PFA 1:25
Anti-human Calretinin Leica Biosystems (NCL-L-CALRET-566) PFA 1:100
Anti-human Cytokeratin Agilent (M3515) PFA 1:100

Secondary
Alexa Fluor 546 goat anti-rat IgG Invitrogen (A-11081) 1:200
Biotinylated goat anti-rat DAKO (E0466) PFA 1:200
Biotinylated goat anti-rabbit Vector laboratories
(BA-1000) PFA 1:200

Conjugated
Alexa Fluor 488 anti-mouse podoplanin eBioscience (53-5381-82) In vivo injection before tissue fixation 5ug intra peritoneal injection
Alexa Fluor 488 anti-mouse alpha smooth muscle actin eBioscience (53-9760-82) PFA 1:200

Supplemental Table 5. Antibodies for Flow Cytometry

Rat Anti-Mouse CD45 30-F11 (BD, #564279) BUV 395 1:200
Rat Anti-Mouse Ly6G RB6-8C5 (Invitrogen, #25-5931-81) PE-Cy7 1:200
Rat Anti-Mouse Ly6C HK1.4 (Invitrogen, #45-5932-80) PerCP-Cy5.5 1:200
Aanti-mouse podoplanin eBioscience (53-5381-82) Alexa Fluor 488 1:200

Supplemental Table 6. Antibodies for Western Blot

Primary
Anti-mouse EGFR D38B1 (Cell signaling #4267) 1:1000
Anti-mouse p-EGFR (Tyr1068) D7A5 (Cell signaling #3777) 1:1000
Anti-mouse Akt Polyclonal (Cell signaling #9272) 1:1000
Anti-mouse p-Akt (Thr308) D25E6 (Cell signaling #13038) 1:1000
Anti-mouse Stat3 124H6 (Cell signaling #9139) 1:1000
Anti-mouse p-Stat3 (Ser727) Polyclonal (Cell signaling #9134) 1:1000
Anti-mouse p44/42 MAPK (Erk1/2) Polyclonal (Cell signaling #9102) 1:1000
Anti-mouse p-p44/42 MAPK (Erk1/2) (Thr202/Tyr204) Polyclonal (Cell signaling #9101) 1:1000
Anti-mouse α-SMA 1A4 (Sigma-Aldrich #A5228) 1:1000
Anti-mouse Vimentin EPR3776 (abcam #ab92547) 1:1000
Anti-mouse E-cadherin 36/E-Cadherin (BD Bioscience #610181) 1:1000

Secondary
Goat anti-Mouse IgG IRDye® 800CW Polyclonal (Licor #925-32210) 1:1000
Goat anti-Rabbit IgG IRDye® 680LT Polyclonal (Licor #925-68021) 1:1000
Goat anti-Rabbit IgG IRDye® 800CW Polyclonal (Licor #925-32211) 1:1000
Goat anti-Rat IgG IRDye® 680LT Polyclonal (Licor #925-68029) 1:1000
Conjugated
HRP-conjugated β-actin AC-15 (Sigma-Aldrich #A3854) 1:50000

Validation

Histology
Rat anti-mouse M6A:, Li, Y., et al. PNAS, 2013 show that this gene and antibody is specific for mesothelium. We validated this by

using cryosections and found that only the mesothelium but not abominal wall expressed this antibody.
Anti-human mesothelin: Isotype control and specificity for mesothelium. Mesothelin was detected in immersion fixed paraffin-embedded sections of human ovarian cancer tissue (https://www.rndsystems.com/products/human-mesothelin-antibody-420404_mab32651).
Anti-human EGFR, anti-human Calretinin, and anti-human Cytokeratin: These antibodies are routinely used as diagnostic markers in the Insitute of Pathology of the University of Bern (accreditation: SN EN ISO/IEC 17025:2018, SN EN ISO 15189:2013). The validation was done according to accredited laboratory guidelines for pathological diagnosis and comprises the validation on at least three positive and negative cases.
Alexa Fluor 488 anti-mouse podoplanin: Several papers, some of the from our lab, (Zindel et. al. Science 2021, Lua et al. J. Hepatol 2016, Wang et al Cell 2016) have shown the specificity of this antibody for mesothelium when injected i.p. For this project we validated the antibody again against isotype control.
Alexa Fluor 488 anti-mouse alpha smooth muscle actin: Validated against isotype control. Cultured mesothelial cells serve as positive control, peritoneal immune cells as negative control.

Flow cytometry:
Rat Anti-Mouse CD45: Validated against Isotype and FMO control, negativ control: mesothelial cells, positive control: peritoneal macrophages.
Rat Anti-Mouse Ly6G: Validated against Isotype and FMO control, negativ control: mesothelial cells, positive control: neutrophils isolated from blood.
Rat Anti-Mouse Ly6C: Validated against Isotype and FMO control, negativ control: mesothelial cells, positive control: bone marrow derived monocytes.

Western blot:
Anti-mouse EGFR: Validated by Hela cells (positive for EGFR in culture) vs. EGFR knock out Hela cells (https://www.cellsignal.com/products/primary-antibodies/egf-receptor-d38b1-xp-rabbit-mab/4267).
Anti-mouse p-EGFR: Validated by comparing BxPC-3 cells, untreated or EGF-stimulated (https://www.cellsignal.com/products/primary-antibodies/phospho-egf-receptor-tyr1068-d7a5-xp-rabbit-mab/3777)
Anti-mouse Akt Polyclonal: CHO cells were transfected with non-targeted (-) or SignalSilence® Akt siRNA I (+) siRNA (https://www.cellsignal.com/products/primary-antibodies/akt-antibody/9272)
Anti-mouse p-Akt: NIH/3T3 cells, untreated (-) or treated with Human Platelet-Derived Growth Factor AA (hPDGF-AA) #8913 (100 ng/ml, 5 min; +), and untreated (-) LNCaP and PC-3 cells (https://www.cellsignal.com/products/primary-antibodies/phospho-akt-thr308-d25e6-xp-rabbit-mab/13038)
Anti-mouse Stat3: Several cell lines (HeLa, NIH/3T3, PC12 and COS cells) are well-documented for baseline Stat3 expression (https://www.cellsignal.com/products/primary-antibodies/stat3-124h6-mouse-mab/9139) and were used as positive controls.
Anti-mouse p-Stat3: A172 cells, untreated (-) or UV-treated (100 mJ, 30 min; +) with or without λ phosphatase (+), using Phospho-Stat3, were used to validate this antibody (https://www.cellsignal.com/products/primary-antibodies/phospho-stat3-ser727-antibody/9134)
Anti-mouse p44/42 MAPK: HeLa cells were transfected with 100 nM control siRNA #6201 (-) or p44 MAPK (Erk1) siRNA (+) (https://www.cellsignal.com/products/primary-antibodies/p44-42-mapk-erk1-2-antibody/9102)
Anti-mouse p-p44/42 MAPK: The antibody reacts specifically with as little as 0.25 ng of phosphorylated p42 MAP kinase and does not cross-react with up to 4 µg of nonphosphorylated p42 MAP kinase (https://www.cellsignal.com/products/primary-antibodies/phospho-p44-42-mapk-erk1-2-thr202-tyr204-antibody/9101)
Anti-mouse α-SMA: This antibody detects smooth muscle actin and labels smooth muscle cells. Uncultured primary mesothelial cells served as negative control.
Anti-mouse Vimentin: HeLa, HEK293, Mouse brain, and Rat brain lysates were used to validate this antibody (https://www.abcam.com/vimentin-antibody-epr3776-cytoskeleton-marker-ab92547.html#lb)
Anti-mouse E-cadherin: A431 (a carcinoma cell line known to express E-cadherin) and 293F cells transfected with E-Cadherin vs. control 293F cell lysates were used to validate this antibody (https://www.bdbiosciences.com/en-us/products/reagents/microscopy-imaging-reagents/immunofluorescence-reagents/purified-mouse-anti-e-cadherin.610182)

Animals and other organisms

Policy information about studies involving animals; ARRIVE guidelines recommended for reporting animal research

Laboratory animals	Female C57BL/6(J) mice with 8 to 12 weeks of age were purchased from Envigo, Netherlands. Animals were housed in specific-pathogen-free (SPF) conditions with free access to water and food,a 12 hour day-night cycle in the central animal facility of the University of Bern, Switzerland. The ambient temperature was 20 +/- 2 degrees Celsius and humidity was kept at 50 +/- 10 %.<br><br>Female Wt1CreERT2 Rosa26tdTomato reporter mice 25 were housed in SPF conditions with free access to water in the central animal facility of the University of Calgary, Canada. The ambient temperature was 21 degrees Celsius, and humidity was kept at 32 %. Female Wt1CreERT2 Rosa26tdTomato reporter were used for experiments at age 10-12 weeks.
Wild animals	no wild animals were used in this study
Field-collected samples	no field-collected samples were used in this study
Ethics oversight	All animal experiments were carried out in accordance with Swiss federal regulations (BE 18/17 and BE 55/18) and in accordance with Canadian legislations and policies (AC19-0148 JZ-PA).

Note that full information on the approval of the study protocol must also be provided in the manuscript.

Human research participants

Policy information about studies involving human research participants

Population characteristics	There are two patient cohorts presented in this study<br>Cohort 1 was a retrospective cohort of patients undergoing surgery for either acute appendicitis or tumor surgery. Details demographics are in Table 2. Patients gave their consent for histological samples to be used for scientific studies prior to surgery.<br>Cohort 2 was a prospective biobanking study that sampled several biological materials of patients that underwent abdominal surgery. Details of patient demographics in Table 3. All patients gave consent prior to surgery.
Recruitment	Cohort 1: Selection of patients was done by the Research Data Support team of the tissue bank of the institute of pathology university of Bern. In brief patients that were treated in our hostpital for acute appendicitis and colorectal cancer were screened for consent and the availability of histological material. Data were anonymized during these steps. For more detail we will attach the study protocol that was accepted by the ethical committee. We have no indication for selection bias. Since every appendectomy specimen is sent to pathology, the current population should reflect the overall population suffering from acute appendicitis despite the fact that this data was collected retrospectively.<br>Cohort 2: All patients presenting in the Bauchzentrum to undergo elective surgery were screened for this study. Detailed inclusion and exclusion criteria are outlined in the study protocol which was accepted by the ethical committee. Patients gave consent for various biopsies including peritoneum and peritoneal fluid. A subgroup of patients in this study had to undergo re-surgery for various reasons (details in the manuscript). In these patients peritoneal fluid and peritoneum (including the attached adhesions) was sampled for the current project. In this prospective study patients were asked whether they want to participate or not. In addition to the samples shown in this study, additional samples were taken in the overarching biobanking study. As such, this comprises a selection bias as patients unwilling to have their samples analyzed would have been less likely to participate. However, we have no indication that this selection bias affects the data presented and conclusions drawn in any way.
Ethics oversight	Kantonale Ethikkommission Bern (KEK), Cohort 1: ID 2020-00077 approved on March 2nd 2020, Cohort 2: ID 2017-00573, Amendment approved on October 10th 2018

Note that full information on the approval of the study protocol must also be provided in the manuscript.

Flow Cytometry

Plots

Confirm that:

- [x] The axis labels state the marker and fluorochrome used (e.g. CD4-FITC).
- [x] The axis scales are clearly visible. Include numbers along axes only for bottom left plot of group (a 'group' is an analysis of identical markers).
- [x] All plots are contour plots with outliers or pseudocolor plots.
- [x] A numerical value for number of cells or percentage (with statistics) is provided.

Methodology

Sample preparation	Mouse peritoneal lavage was performed in anesthetized animals by triple lavage with sterile ice cold 2 mM EDTA containing PBS into the peritoneal cavity and subsequent retrieval. Lavage samples were subsequently centrifuged at 500g for 5 min at 4°C and cell pellet was subsequently processed for flow cytometry. The cells were incubated in protein based live/dead stain and blocked using anti-mouse CD16/32 antibody (2.4G2 clone, BioXcell) for 20 min. Cells were then stained for 20 min with specified markers as described in paragraph of antibodies.
Instrument	Samples were run using BD FACS Canto or BD FACS Fortessa flow cytometer
Software	Events were analyzed using FlowJo software (Tree Star)
Cell population abundance	The post-sort purity of peritoneal leukocyte fractions was assessed by re-running flow cytometry with the same gating for each targeted population with a targeted purity of > 90%.
Gating strategy	A representative example is shown in Figure S11.

- [x] Tick this box to confirm that a figure exemplifying the gating strategy is provided in the Supplementary Information.
